# The impact of filgotinib on patient-reported outcomes and health-related quality of life for patients with active rheumatoid arthritis: a post hoc analysis of Phase 3 studies

**DOI:** 10.1186/s13075-021-02677-7

**Published:** 2022-01-03

**Authors:** Clifton O. Bingham, David Walker, Peter Nash, Susan J. Lee, Lei Ye, Hao Hu, Javaria Mona Khalid, Bernard Combe

**Affiliations:** 1 Division of Rheumatology, Johns Hopkins University, 5200 Eastern Avenue, Mason F. Lord Bldg, Center Tower, Room 434A, Baltimore, MD 21224 USA; 2 Northumbria Healthcare Trust, North Shields, UK; 3 Griffith University, Brisbane, QLD Australia; 4 Gilead Sciences, Inc., Foster City, CA USA; 5 Galapagos BV, Leiden, Netherlands; 6 University of Montpellier, Montpellier, France

**Keywords:** Filgotinib, Patient-reported outcomes, HAQ-DI, SF-36, FACIT-Fatigue, WPAI

## Abstract

**Background:**

The effects of filgotinib on patient-reported outcomes (PROs) from 3 trials in patients with active rheumatoid arthritis were investigated.

**Methods:**

Methotrexate (MTX)-naïve patients received filgotinib 200 or 100 mg plus MTX (FIL200+MTX, FIL100+MTX), filgotinib 200 mg monotherapy (FIL200), or MTX monotherapy through 52 weeks (NCT02886728). Patients with inadequate response (IR) to MTX (MTX-IR) received FIL200+MTX, FIL100+MTX, adalimumab 40 mg +MTX (ADA+MTX), or placebo (PBO)+MTX (rerandomized to FIL200+MTX or FIL100+MTX at week 24) through 52 weeks (NCT02889796). Patients with IR to biologic disease-modifying antirheumatic drugs (bDMARD-IR) received FIL200 or FIL100 or PBO with background stable conventional synthetic (cs) DMARDs for up to 24 weeks (NCT02873936). PROs included Health Assessment Questionnaire-Disability Index (HAQ-DI), Medical Outcomes Study 36-Item Short Form Health Survey (SF-36) physical/mental component summary (PCS/MCS), Functional Assessment of Chronic Illness Therapy-Fatigue (FACIT-Fatigue), Work Productivity and Activity Impairment Questionnaire-Rheumatoid Arthritis (WPAI-RA), and Patient Global Assessment of Disease Activity (PtGA). Data are reported as least-squares mean changes from baseline with standard error to the timepoint representing each study’s primary endpoint. All statistical comparisons are of filgotinib groups vs their respective control groups.

**Results:**

At week 24, among MTX-naïve patients, change from baseline (standard deviation) in HAQ-DI was − 1.00 (0.03; *P* < 0.001) with FIL200+MTX, − 0.94 (0.04; *P* < 0.01) with FIL100+MTX, and − 0.91 (0.04; *P* < 0.05) with FIL200 alone compared with − 0.81 (0.03) with MTX alone. At week 12, among MTX-IR patients, change from baseline in HAQ-DI was − 0.69 (0.04; *P* < 0.001 vs PBO+MTX, *P* < 0.05 vs ADA) with FIL200+MTX, − 0.57 (0.04; *P* < 0.001 vs placebo) with FIL100+MTX, and − 0.60 (0.04) with ADA vs − 0.40 (0.04) with PBO+MTX. At week 12, among bDMARD-IR patients, change from baseline in HAQ-DI was − 0.50 (0.06; *P* < 0.001) with FIL200+csDMARD and − 0.46 (0.05; *P* < 0.001) with FIL100+csDMARD vs − 0.19 (0.06) with placebo+csDMARD. Changes in SF-36 PCS and MCS, FACIT-Fatigue, WPAI, and PtGA tended to favor filgotinib over PBO, MTX, and ADA. Greater proportions of patients experienced clinically meaningful differences with either dosage of FIL in combination with csDMARDs (including MTX) and with FIL200 monotherapy vs comparators.

**Conclusions:**

Filgotinib provided improvements in PROs across patient populations. These findings suggest filgotinib can be an effective treatment option for patients with insufficient response to MTX or bDMARDs and patients who are MTX-naïve.

**Trial registration:**

ClinicalTrials.gov, FINCH 1, NCT02889796, first posted September 7, 2016; FINCH 2, NCT02873936, first posted August 22, 2016, retrospectively registered; FINCH 3, NCT02886728, first posted September 1, 2016, retrospectively registered.

**Supplementary Information:**

The online version contains supplementary material available at 10.1186/s13075-021-02677-7.

## Introduction

Rheumatoid arthritis (RA) is an autoimmune disease in which immunologically driven inflammation leads to deterioration and impairment of joint tissue [1]. Functional impairment and chronic pain resulting from inflammation and progressive joint damage are common among patients with RA [1, 2]. This impairment and pain often lead to disability; loss of joint function; inability to participate in desired family, social, and leisure activities; and reduced productivity at work, all of which negatively impact health-related quality of life (HRQL) [1, 2]. HRQL assessments have become an important part of many clinical RA studies to better evaluate the patient experience and the impact of treatment on patient well-being [3, 4].

Filgotinib is a once-daily, oral, Janus kinase 1 (JAK1) preferential inhibitor [5]. In three Phase 3 studies, filgotinib improved signs and symptoms of RA and physical function in patients with inadequate response to methotrexate (MTX-IR; FINCH 1, NCT02889796) or biologic disease-modifying antirheumatic drugs (bDMARD-IR; FINCH 2, NCT02873936), and in patients with early RA who had not been treated with MTX (MTX-naïve; FINCH 3, NCT02886728) [1, 6, 7]. In these studies, higher proportions of patients achieved the primary endpoint of 20% improvement in American College of Rheumatology (ACR20) criteria relative to placebo. The present analysis evaluated the effect of filgotinib on measures of HRQL in patients with RA by analyzing patient-reported outcomes (PROs) captured during these clinical trials, including the Health Assessment Questionnaire-Disability Index (HAQ-DI), Medical Outcomes Study 36-Item Short Form Survey (SF-36), Functional Assessment of Chronic Illness Therapy-Fatigue (FACIT-Fatigue), Work Productivity and Activity Impairment Questionnaire-Rheumatoid Arthritis (WPAI-RA), and Patient Global Assessment of Disease Activity (PtGA).

## Methods

### Study design

All 3 trials were randomized, double-blind, placebo- (MTX-IR, bDMARD-IR) or active-controlled (adalimumab for the MTX-IR trial and MTX for the MTX-naïve trial), multicenter, Phase 3 studies. The trials were conducted in accordance with the Declaration of Helsinki and International Council for Harmonisation Good Clinical Practice guidelines. The study protocols were approved by the international review board or ethics committee at each study site, and all patients provided written informed consent. This is a post hoc analysis of PROs.

Details of the studies have been reported previously [1, 6, 7]. Patients were aged ≥ 18 years with ≥ 6 swollen joints (from swollen joint count of 66 joints) and ≥ 6 tender joints (from tender joint count of 68 joints) at screening and baseline. MTX-naïve patients had limited or no prior treatment with MTX (no more than 3 doses of MTX ≤ 25 mg each in the patient’s lifetime for the treatment of RA), with the last dose at least 28 days prior to study day 1. Patients in this trial were randomized 2:1:1:2 to filgotinib 200 mg plus MTX, filgotinib 100 mg plus MTX, filgotinib 200 mg monotherapy, or MTX monotherapy administered for up to 52 weeks. The time point for the primary endpoint—the proportion of patients who achieved ACR20 response—was week 24. In the MTX-IR trial, patients receiving ongoing MTX treatment for ≥ 12 weeks at a dose of 7.5 to 25 mg/week were randomized 3:3:2:3 to receive once daily filgotinib 200 mg, filgotinib 100 mg, subcutaneous adalimumab 40 mg, or placebo for up to 52 weeks with a stable weekly background of MTX. At week 24, patients receiving placebo who did not discontinue treatment were rerandomized 1:1 to filgotinib 200 or 100 mg. The time point for the primary endpoint—the proportion of patients who achieved ACR20 response—was week 12. Patients in the bDMARD-IR trial were randomized 1:1:1 to receive once-daily filgotinib 200 mg, filgotinib 100 mg, or placebo for up to 24 weeks, all on background stable dosages of conventional synthetic (cs) DMARDs. The time point for the primary endpoint—the proportion of patients who achieved ACR20 response—was week 12.

### Patient-reported outcomes

HAQ-DI, SF-36, FACIT-Fatigue, WPAI-RA, and PtGA were collected prospectively throughout the studies. HAQ-DI assesses 8 functional categories (dressing and grooming, arising, eating, walking, hygiene, reach, grip, and other activities), with scores ranging from 0 (no disability) to 3 (completely disabled) [8–10]. The SF-36 is a 36-item questionnaire grouped into 8 scales, which can be further summarized as Physical and Mental Component scores; scores range from 0 to 100, representing “least health” to “greatest health.” [11] FACIT-Fatigue is a 13-item questionnaire concerning fatigue severity and impact over the previous week that is scored from 0 to 52, with higher scores indicating less fatigue [11]. WPAI is a quantitative assessment of the amount of absenteeism, presenteeism, and daily activity impairment attributable to general health or a specific health problem [12, 13]. The RA-specific version, WPAI-RA, is a self-administered 6-item questionnaire; activity impairment is a single item score evaluated among all patients, while work productivity impairment (employed patients only) is a weighted score of absenteeism and presenteeism. Negative values represent improvement [14]. PtGA uses a visual analog scale ranging from 0 to 100 mm; higher scores indicate worse disease [1]. These questionnaires have been validated and used extensively in RA clinical trials [8–11, 14–18].

In all three Phase 3 studies, HAQ-DI and PtGA were assessed at baseline and at weeks 2, 4, 8, 12, 16, 20, and 24. In the MTX-IR and MTX-naïve trials, assessments were also made at weeks 30, 36, 44, and 52. SF-36, FACIT-Fatigue, and WPAI-RA were collected at baseline and weeks 4, 12, and 24, as well as weeks 36 and 52 for the MTX-IR and MTX-naïve trials. The minimally clinically important difference (MCID) was defined as achievement of a ≥ 0.22 point reduction from baseline for HAQ-DI, a ≥ 2.5-point increase from baseline for SF-36 Mental Component Score (MCS) and Physical Component Score (PCS), and a ≥ 4-point increase from baseline for FACIT-Fatigue [11, 19, 20].

### Statistical analyses

Mean changes from baseline in HAQ-DI, SF-36, FACIT-Fatigue, WPAI, and PtGA for each filgotinib arm were compared with mean changes from baseline in placebo (MTX-IR and bDMARD-IR trials), adalimumab (MTX-IR trial), or MTX (MTX-naïve trial) using the mixed-effects model for repeated measures (MMRM), including treatment group, visit, treatment group by visit interaction, stratification factors (including geographic region, presence of rheumatoid factor or anti-cyclic citrullinated peptide antibodies, and prior exposure to bDMARDs), and baseline value of the measure being tested as fixed effects, and patients as random effect. Missing change scores due to missing study visits or early withdrawal were not otherwise imputed using the MMRM approach.

Additional analyses included the proportion of patients with HAQ-DI ≤ 0.5 and the proportion of patients achieving HAQ-DI, SF-36, and FACIT-Fatigue MCID. The proportion of patients achieving these thresholds with each filgotinib regimen and with placebo (MTX-IR and bDMARD-IR trials), adalimumab (MTX-IR), or MTX (MTX-naïve) were compared using a logistic regression model including treatment group and stratification factors. A nonresponder imputation was used for patients with missing data. All analyses were exploratory without multiplicity adjustment, and exploratory *P* values are reported.

## Results

### Baseline demographics and disease characteristics

Of the patients randomized and treated, 1025/1249 (82.1%) MTX-naïve patients, 1517/1755 (86.4%) MTX-IR patients, and 381/448 (85.0%) bDMARD-IR patients completed their studies. Patient demographics and baseline disease characteristics were comparable between the treatment groups in each study and are listed in Supplemental Tables 1, 2 and 3. Baseline PRO scores were similar between treatment groups in each study and indicated a substantial disease burden (Supplemental Tables 1, 2 and 3).

### Differences between treatment groups in HAQ-DI changes from baseline

At week 4, improvements in HAQ-DI score from baseline were greater for MTX-naïve patients who received filgotinib 200 mg plus MTX (− 0.64 ± 0.03), filgotinib 100 mg plus MTX (− 0.50 ± 0.04), or filgotinib 200 mg monotherapy (− 0.56 ± 0.04) than with MTX alone (− 0.35 ± 0.03, *P* < 0.001 for all filgotinib treatments vs MTX alone) (Table 1). Improvements were significantly greater for those receiving any dose or treatment regimen of filgotinib relative to MTX alone at week 24, and improvements remained greater for filgotinib 200 mg plus MTX and filgotinib 200 mg monotherapy compared to MTX alone at week 52. Improvements for patients receiving filgotinib 100 mg plus MTX and MTX alone were similar at week 52.

**Table 1:** Least-squares mean change from baseline for patient-reported outcomes in MTX-naïve patients; filgotinib dosing regimens compared to MTX

PRO measures	FIL 200 mg + MTX ***n*** = 416	FIL 100 mg + MTX ***n*** = 207	FIL 200 mg ***n*** = 210	MTX ***n*** = 416
**HAQ-DI**				
Week 4	− 0.64 (0.03)^***^	− 0.50 (0.04)^***^	− 0.56 (0.04)^***^	− 0.35 (0.03)
Week 12	− 0.91 (0.03)^***^	− 0.82 (0.04)^***^	− 0.80 (0.04)^***^	− 0.64 (0.03)
Week 24	− 1.00 (0.03)^***^	− 0.94 (0.04)^**^	− 0.91 (0.04)^*^	− 0.81 (0.03)
Week 52	− 1.03 (0.04)^***^	− 0.96 (0.05)	− 0.98 (0.05)^*^	− 0.87 (0.04)
**SF-36 PCS**				
Week 4	7.3 (0.4)^***^	5.8 (0.5)^**^	6.4 (0.5)^***^	4.1 (0.4)
Week 12	11.6 (0.4)^***^	9.6 (0.6)^**^	9.4 (0.6)^*^	7.9 (0.4)
Week 24	12.8 (0.5)^***^	11.5 (0.6)^*^	10.8 (0.6)	9.9 (0.5)
Week 52	13.6 (0.5)^***^	12.0 (0.7)	12.2 (0.7)	10.8 (0.5)
**SF-36 MCS**				
Week 4	4.6 (0.5)^***^	3.4 (0.6)^*^	4.3 (0.6)^***^	1.9 (0.5)
Week 12	5.7 (0.5)^*^	5.7 (0.7)	5.4 (0.7)	4.3 (0.5)
Week 24	5.9 (0.5)	6.3 (0.7)	5.5 (0.7)	5.7 (0.5)
Week 52	6.5 (0.5)	6.2 (0.7)	5.8 (0.7)	5.8 (0.6)
**FACIT-Fatigue**				
Week 4	7.6 (0.5)^***^	6.9 (0.6)^***^	7.1 (0.6)^***^	4.4 (0.5)
Week 12	10.3 (0.5)^***^	9.3 (0.7)	9.8 (0.7)^*^	8.1 (0.5)
Week 24	11.3 (0.6)	11.3 (0.7)	10.3 (0.7)	10.0 (0.6)
Week 52	12.1 (0.6)^*^	11.4 (0.8)	11.6 (0.8)	10.4 (0.6)
**PtGA**				
Week 4	− 28 (1.2)^***^	− 21 (1.6)^**^	− 23 (1.6)^***^	− 15 (1.2)
Week 12	− 38 (1.3)^***^	− 31 (1.7)^**^	− 32 (1.7)^**^	− 26 (1.3)
Week 24	− 44 (1.3)^***^	− 38 (1.7)	− 38 (1.7)	− 34 (1.3)
Week 52	− 46 (1.4)^***^	− 40 (1.8)	− 43 (1.8)^**^	− 37 (1.4)
**WPAI-RA**				
**Absenteeism**				
Week 4	− 2.9 (2.0)	− 0.3 (2.8)	− 4.8 (2.7)	− 0.2 (2.1)
Week 12	− 6.2 (1.9)	− 8.3 (2.6)	− 2.9 (2.4)	− 3.8 (1.8)
Week 24	− 6.7 (1.7)	− 10.2 (2.3)	− 3.6 (2.1)^*^	− 9.0 (1.7)
Week 52	− 7.0 (1.8)	− 7.9 (2.5)	− 4.2 (2.4)	− 7.6 (1.9)
**Presenteeism**				
Week 4	− 19.4 (2.0)^***^	− 22.3 (2.8)^***^	− 17.3 (2.6)^**^	− 7.2 (2.1)
Week 12	− 27.2 (2.0) ^**^	− 28.2 (2.8)^*^	− 25.4 (2.6)	− 19.9 (2.0)
Week 24	− 29.8 (1.9)	− 32.2 (2.7)	− 27.5 (2.5)	− 26.8 (2.0)
Week 52	− 33.6 (1.9)	− 30.7 (2.7)	− 32.2 (2.5)	− 30.0 (2.1)
**Work productivity loss**				
Week 4	− 19.0 (2.1)^***^	− 21.8 (3.0)^***^	− 17.1 (2.8)^**^	− 6.6 (2.2)
Week 12	− 27.6 (2.2)^**^	− 28.1 (3.1)^*^	− 23.4 (2.9)	− 19.5 (2.2)
Week 24	− 30.3 (2.1)	− 30.5 (3.0)	− 24.6 (2.8)	− 26.4 (2.2)
Week 52	− 33.4 (2.2)	− 31.2 (3.1)	− 29.8 (2.9)	− 30.3 (2.4)
**Activity impairment**				
Week 4	− 23.2 (1.3)^***^	− 17.8 (1.7)^*^	− 19.7 (1.7)^**^	− 13.5 (1.3)
Week 12	− 32.5 (1.4)^***^	− 28.1 (1.8)^*^	− 30.5 (1.8)^***^	− 23.6 (1.4)
Week 24	− 36.4 (1.4)^*^	− 34.6 (1.8)	− 32.7 (1.8)	− 32.3 (1.4)
Week 52	− 39.9 (1.4)^**^	− 36.4 (1.9)	− 38.4 (1.9)	− 34.5 (1.5)

Data presented as LS mean (SE). The primary endpoint was assessed at Week 24. Comparison with methotrexate: ^***^P < 0.001, ^**^P < 0.01, ^*^P < 0.05. All *P* values are exploratory (not adjusted for multiplicity) and were assessed using the MMRM, including treatment group, visit, treatment group by visit interaction, stratification factors, and baseline value as fixed effects, and patients as random effect.

*FACIT*, Functional Assessment of Chronic Illness Therapy; *FIL*, filgotinib; *HAQ-DI*, Health Assessment Questionnaire-Disability Index; *LS*, least-squares; *MCS*, Mental Component Score; *MTX*, methotrexate; *PCS*, Physical Component Score; *PRO*, patient-reported outcomes; *PtGA*, Patient Global Assessment of Disease Activity; *SE*, standard error; *SF-36*, Medical Outcomes Study 36-Item Short Form; *WPAI-RA*, Work Productivity and Activity Impairment Questionnaire-Rheumatoid Arthritis

Among MTX-IR patients, improvements in HAQ-DI were greater at weeks 4, 12, and 24 for patients receiving filgotinib 200 mg plus MTX and 100 mg plus MTX compared to placebo. Improvements in HAQ-DI at week 4 were − 0.44 ± 0.04 for patients receiving filgotinib 200 mg and − 0.35 ± 0.04 for patients receiving filgotinib 100 mg compared to − 0.25 ± 0.04 for placebo (*P* < 0.001 for both) (Table 2). HAQ-DI improvements were similar for patients receiving filgotinib 200 mg plus MTX at weeks 4 and 24 relative to adalimumab plus MTX, while improvements from baseline were greater among patients who received filgotinib 200 mg plus MTX relative to adalimumab plus MTX at weeks 12 and 52 (Table 2). For filgotinib 100 mg plus MTX, improvements from baseline were similar throughout the study compared with patients receiving adalimumab plus MTX.

**Table 2:** Least-squares mean change from baseline for patient-reported outcomes in MTX-IR patients; filgotinib dosing regimens compared to placebo and adalimumab

PRO measures	FIL 200 mg + MTX ***n*** = 475	FIL 100 mg + MTX ***n*** = 480	ADA +MTX ***n*** = 325	PBO ***n*** = 475
**HAQ-DI**				
Week 4	− 0.44 (0.04)^***^	− 0.35 (0.04)^***^	− 0.40 (0.04)	− 0.25 (0.04)
Week 12	− 0.69 (0.04)^***†^	− 0.57(0.04)^***^	− 0.60 (0.04)	− 0.40 (0.04)
Week 24	− 0.82 (0.04)^***^	− 0.74 (0.04)^***^	− 0.76 (0.04)	− 0.55 (0.04)
Week 52	− 0.90 (0.04)^†^	− 0.82 (0.04)	− 0.82 (0.05)	–
**SF-36 PCS**				
Week 4	6.0 (0.5)^***†^	5.1 (0.5)^***^	5.1 (0.6)	3.4 (0.6)
Week 12	9.7 (0.6)^***†^	9.1 (0.6)^***^	8.6 (0.6)	5.9 (0.6)
Week 24	10.7 (0.6)^***^	10.6 (0.6)^***^	10.3 (0.6)	7.6 (0.6)
Week 52	12.0 (0.6)	11.6 (0.6)	11.9 (0.7)	–
**SF-36 MCS**				
Week 4	3.3 (0.6)^***^	3.0 (0.6)^**^	3.2 (0.7)	1.5 (0.6)
Week 12	4.8 (0.6)^**^	5.0 (0.6)^**^	4.2 (0.7)	3.3 (0.6)
Week 24	5.4 (0.6)	5.3 (0.6)	4.7 (0.7)	4.4 (0.7)
Week 52	5.7 (0.7)	6.2 (0.7)	5.5 (0.7)	–
**FACIT-Fatigue**				
Week 4	6.5 (0.7)^***^	5.9 (0.7)^***^	5.7 (0.7)	3.7 (0.7)
Week 12	9.4 (0.7)^***^	9.3 (0.7)^***^	8.8 (0.7)	6.6 (0.7)
Week 24	10.7 (0.7)^***^	10.8 (0.7)^***^	10.0 (0.8)	8.0 (0.7)
Week 52	11.7 (0.7)	11.8 (0.7)	10.9 (0.8)	–
**PtGA**				
Week 4	− 23 (1.5)^***^	− 18 (1.5)^***^	− 21 (1.6)	− 14 (1.6)
Week 12	− 34 (1.6)^***†^	− 30 (1.6)^***^	− 29 (1.8)	− 21 (1.6)
Week 24	− 40 (1.6)^***^	− 37 (1.6)^***^	− 37 (1.8)	− 29 (1.7)
Week 52	− 43 (1.7)	− 40 (1.7)	− 41 (1.9)	–
**WPAI-RA**				
**Absenteeism**				
Week 4	0.6 (2.1)	− 1.3 (2.1)	− 0.9 (2.4)	− 0.4 (2.2)
Week 12	− 2.3 (2.0)	− 2.7 (2.0)	− 2.1 (2.3)	− 0.2 (2.2)
Week 24	− 3.0 (2.0)^**^	− 3.5 (2.0)^**^	− 2.3 (2.3)	2.2 (2.2)
Week 52	− 1.5 (1.8)	− 0.0 (1.9)	0.6 (2.2)	–
**Presenteeism**				
Week 4	− 13.1 (2.6)^**^	− 9.0 (2.5)	− 11.4 (2.9)	− 6.1 (2.7)
Week 12	− 20.9 (2.6)^***^	− 20.2 (2.5)^**^	− 20.3 (2.9)	− 13.0 (2.8)
Week 24	− 25.2 (2.6)^***^	− 23.8 (2.6)^**^	− 21.0 (3.0)	− 16.3 (2.9)
Week 52	− 28.6 (2.5)	− 27.0 (2.5)	− 25.3 (2.9)	–
**Work productivity loss**				
Week 4	− 11.7 (2.8)^**^	− 7.9 (2.7)	− 10.9 (3.1)	− 5.3 (2.9)
Week 12	− 19.3 (2.8)^**^	− 19.2 (2.7)^**^	− 18.5 (3.2)	− 11.6 (3.0)
Week 24	− 23.8 (2.9)^***^	− 22.9 (2.8)^***^	− 19.2 (3.3)	− 13.4 (3.1)
Week 52	− 26.8 (2.8)	− 24.4 (2.8)	− 22.7 (3.2)	–
**Activity impairment**				
Week 4	− 18.0 (1.7)^***^	− 16.1 (1.7)^***^	− 16.0 (1.8)	− 10.6 (1.7)
Week 12	− 27.3 (1.7)^***†^	− 25.6 (1.7)^***^	− 23.7 (1.9)	− 17.9 (1.8)
Week 24	− 31.5 (1.7)^***^	− 31.3 (1.8)^***^	− 29.2 (1.9)	− 22.0 (1.8)
Week 52	− 35.4 (1.8)	− 35.1 (1.8)	− 33.1 (2.0)	–

Data presented as LS mean (SE). The primary endpoint was assessed at Week 12. Comparison with placebo: ^***^P < 0.001, ^**^P < 0.01. Comparison with adalimumab: ^†^*P* < 0.05. All *P* values are exploratory (not adjusted for multiplicity) and assessed using the MMRM, including treatment group, visit, treatment group by visit interaction, stratification factors, baseline value as fixed effects, and patients as random effect. In FINCH 1, patients on PBO were rerandomized to FIL 200 or 100 mg at week 24.

*ADA*, adalimumab; *FACIT*, Functional Assessment of Chronic Illness Therapy; *FIL*, filgotinib; *LS*, least-squares; *HAQ-DI*, Health Assessment Questionnaire-Disability Index; *MCS*, Mental Component Score; *MTX*, methotrexate; *PBO*, placebo; *PCS*, Physical Component Score; *PRO*, patient-reported outcomes; *PtGA*, Patient Global Assessment of Disease Activity; *SE*, standard error; *SF-36*, Medical Outcomes Study 36-Item Short Form; *WPAI-RA*, Work Productivity and Activity Impairment Questionnaire-Rheumatoid Arthritis

Patients who were bDMARD-IR and on a background of csDMARDs who received filgotinib 200 (− 0.35 ± 0.05) and 100 mg (− 0.28 ± 0.05) reported improvements relative to placebo (− 0.13 ± 0.05, *P* < 0.001 and *P* = 0.006) at week 4. Significant improvements were maintained through week 24 (Table 3).

**Table 3:** Least-squares mean change from baseline for patient-reported outcomes in bDMARD-IR patients; filgotinib dosing regimens compared to placebo

PRO measures	FIL 200 mg + csDMARD ***n*** = 147	FIL 100 mg + csDMARD ***n *** = 153	PBO + csDMARD ***n*** = 148
**HAQ-DI**			
Week 4	− 0.35 (0.05)^***^	− 0.28 (0.05)^**^	− 0.13 (0.05)
Week 12	− 0.50 (0.06)^***^	− 0.46 (0.05)^***^	− 0.19 (0.06)
Week 24	− 0.63 (0.06) ^***^	− 0.49 (0.06)^**^	− 0.27 (0.06)
**SF-36 PCS **			
Week 4	5.9 (0.7) ^***^	5.4 (0.7)^**^	3.4 (0.7)
Week 12	8.4 (0.8)^***^	7.6 (0.8)^***^	4.2 (0.8)
Week 24	9.6 (0.8)^***^	8.9 (0.8)^**^	5.8 (0.9)
**SF-36 MCS**			
Week 4	2.5 (0.9)^*^	2.0 (0.9)	0.3 (0.9)
Week 12	4.5 (0.9) ^*^	3.5 (0.9)	2.5 (0.9)
Week 24	4.9 (1.0)	3.1 (1.0)	3.0 (1.1)
**FACIT-Fatigue **			
Week 4	6.9 (1.0) ^***^	6.6 (1.0)^**^	3.3 (1.0)
Week 12	10.2 (1.0)^***^	8.4 (1.0)^**^	5.2 (1.1)
Week 24	11.5 (1.1)^***^	9.0 (1.1)	6.9 (1.2)
**PtGA **			
Week 4	− 21 (2.3) ^***^	− 20 (2.3)^***^	− 9 (2.4)
Week 12	− 31 (2.5)^***^	− 26 (2.5)^***^	− 13 (2.6)
Week 24	− 36 (2.6)^***^	− 30 (2.7)^***^	− 18 (2.8)
**WPAI-RA **			
**Absenteeism **			
Week 4	− 5.9 (3.5)	− 1.3 (2.9)	2.4 (3.0)
Week 12	− 6.9 (3.6)^*^	− 3.0 (3.0)	3.1 (3.2)
Week 24	− 5.7 (4.4)	− 0.5 (3.9)	4.1 (4.5)
**Presenteeism **			
Week 4	− 22.0 (3.9) ^***^	− 12.5 (3.3)	− 3.1 (3.5)
Week 12	− 23.5 (4.1)^**^	− 18.0 (3.5)^*^	− 6.8 (3.6)
Week 24	− 20.9 (4.0)	− 24.6 (3.7)	− 15.1 (4.0)
**Work productivity loss **			
Week 4	− 22.9 (4.1) ^***^	− 11.3 (3.5)	− 2.7 (3.7)
Week 12	− 25.8 (4.4)^***^	− 17.7 (3.7)^*^	− 5.7 (3.9)
Week 24	− 21.7 (4.6)	− 22.8 (4.3)	− 12.8 (4.7)
**Activity impairment **			
Week 4	− 15.9 (1.9) ^***^	− 14.2 (1.9)^***^	− 4.6 (1.9)
Week 12	− 24.4 (2.1) ^***^	− 18.8 (2.1)^**^	− 10.5 (2.1)
Week 24	− 30.1 (2.2) ^***^	− 24.4 (2.3)^**^	− 15.4 (2.4)

Data presented as LS mean (SE). The primary endpoint was assessed at Week 12. Comparison with placebo: ^***^P < 0.001, ^**^P < 0.01, ^*^P < 0.05. All *P * values are exploratory (not adjusted for multiplicity) and assessed using the MMRM, including treatment group, visit, treatment group by visit interaction, stratification factors, baseline value as fixed effects, and patients as random effect. The FINCH 2 study ended at week 24.

*csDMARD*, conventional synthetic disease-modifying antirheumatic drug; *FACIT*, Functional Assessment of Chronic Illness Therapy; *FIL*, filgotinib; * LS*, least-squares; *HAQ-DI*, Health Assessment Questionnaire-Disability Index; *MCS *, Mental Component Score; *PBO*, placebo; *PCS*, Physical Component Score; *PRO*, patient-reported outcomes; *PtGA *, Patient Global Assessment of Disease Activity; *SE*, standard error; *SF-36 *, Medical Outcomes Study 36-Item Short Form; *WPAI-RA*, Work Productivity and Activity Impairment Questionnaire-Rheumatoid Arthritis

### Differences between treatment groups in proportions of patients achieving HAQ-DI MCID

**Fig. 1:**
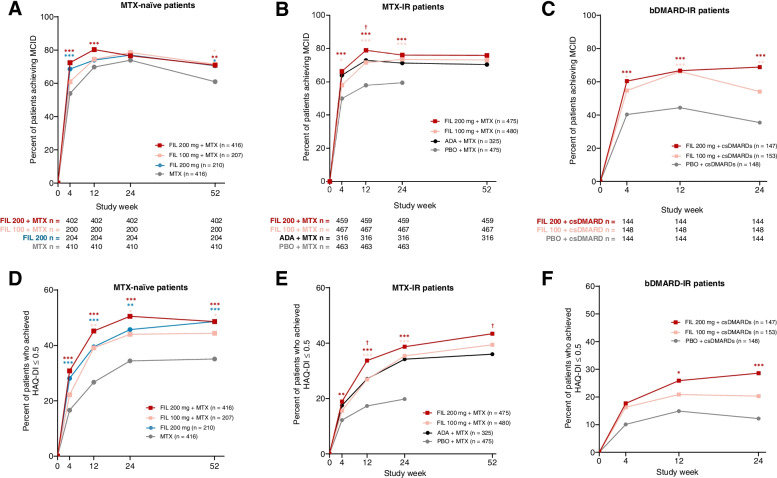
Proportion of patients achieving MCID for HAQ-DI in **A** MTX-naïve patients, **B** MTX-IR patients, and **C** and bDMARD-IR patients; proportion of patients who achieved HAQ-DI score of ≤ 0.5 by visit **D** MTX-naïve, **E** MTX-IR, and **F** bDMARD-IR. Comparison with PBO or MTX: ^***^P < 0.001, ^**^P < 0.01, ^*^P < 0.05. Comparison with ADA: ^†^*P* < 0.05. All *P* values are exploratory (not adjusted for multiplicity) and were from logistic regression with treatment groups and stratification factors in the model. A nonresponder imputation was used for patients with missing data. MCID was defined as a ≥ 0.22-point reduction from baseline. In the MTX-IR trial, patients on PBO were rerandomized to FIL 200 or 100 mg at week 24. The study in bDMARD-IR patients ended at week 24. ADA, adalimumab; bDMARD, biologic DMARD; csDMARD, conventional synthetic DMARD; DMARD, disease-modifying antirheumatic drug; FIL, filgotinib; HAQ-DI, Health Assessment Questionnaire-Disability Index; LS, least-squares; MCID, minimal clinically important difference; MTX, methotrexate; PBO, placebo

The proportion of patients who achieved MCID for HAQ-DI—a ≥ 0.22-point reduction from baseline—increased starting at week 4 in all patient populations. Among MTX-naïve patients at week 4, 72.4% who received filgotinib 200 mg plus MTX (*P* < 0.001), 61.0% who received filgotinib 100 mg plus MTX (*P* = 0.083), and 68.6% who received filgotinib 200 mg (*P* < 0.001) achieved MCID compared to 53.9% of patients who received MTX alone. At week 24, the proportion achieving MCID was similar between patients receiving any dosing regimen of filgotinib and patients receiving MTX alone. Greater proportions of patients who received any dosage of filgotinib achieved MCID relative to those who received MTX monotherapy at week 52 (Fig. 1A).

At week 4, 66.2% of MTX-IR patients who received filgotinib 200 mg (*P* < 0.001) and 58.0% of those who received filgotinib 100 mg (*P* = 0.011) achieved MCID compared to 49.9% of patients who received placebo (Fig. 1B). At weeks 12 and 24, the proportion of MTX-IR patients achieving MCID remained significantly greater for patients receiving either dose of filgotinib relative to placebo. A greater proportion of MTX-IR patients who received filgotinib 200 mg achieved MCID at week 12 than those who received adalimumab (78.9% vs 72.8%, *P* = 0.046); the proportion remained numerically greater through week 52 (Fig. 1B). The percentage of MTX-IR patients achieving MCID for HAQ-DI was numerically greater for patients who received filgotinib 100 mg relative to adalimumab throughout the study (Fig. 1B).

At all timepoints, a greater proportion of bDMARD-IR patients who received filgotinib 200 mg (*P* < 0.001) and filgotinib 100 mg (*P* ≤ 0.013) achieved MCID relative to placebo (Fig. 1C). At week 4, 60.4% of bDMARD-IR patients who received filgotinib 200 mg (*P* < 0.001) and 54.7% who received filgotinib 100 mg (*P* = 0.013) achieved MCID compared to 40.3% of the placebo group.

### Differences between treatment groups in proportions of patients achieving HAQ-DI near normal

The proportion of MTX-naïve patients with HAQ-DI scores of ≤ 0.5 at week 4 was 30.8% (*P* < 0.001) for patients who received filgotinib 200 mg plus MTX, 22.2% (*P* = 0.075) for filgotinib 100 mg plus MTX, and 28.1% (*P* < 0.001) for filgotinib 200 mg monotherapy compared with 16.6% for MTX monotherapy (Fig. 1D). The proportion of patients with HAQ-DI ≤ 0.5 remained greater for patients receiving any dose of filgotinib relative to MTX alone through week 52.

Among MTX-IR patients, HAQ-DI ≤ 0.5 was achieved at week 4 by 18.9% (*P* = 0.004) of patients who received filgotinib 200 mg, 15.6% (*P* = 0.12) receiving filgotinib 100 mg, and 12.2% receiving placebo (Fig. 1E). The proportion of bDMARD-IR patients who achieved HAQ-DI ≤ 0.5 at week 4 was 17.7% (*P* = 0.051) for filgotinib 200 mg, 16.3% (*P* = 0.10) for filgotinib 100 mg, and 10.1% for placebo. These proportions increased at week 12 for patients who received filgotinib 200 mg, filgotinib 100 mg, and placebo (Fig. 1F). This effect was maintained at week 24 for both MTX-IR and bDMARD-IR patients receiving either dose of filgotinib relative to placebo (Fig. 1E, F). The proportion of MTX-IR patients who achieved HAQ-DI ≤ 0.5 was similar for patients receiving either dose of filgotinib and adalimumab at week 4. A greater proportion of patients who received filgotinib 200 mg achieved HAQ-DI ≤ 0.5 compared with adalimumab at weeks 12 and 52. Proportions of patients achieving HAQ-DI ≤ 0.5 were similar for filgotinib 100 mg and adalimumab (Fig. 1E).

### Differences between treatment groups in SF-36 changes from baseline

By week 4, MTX-naïve patients who received filgotinib 200 mg plus MTX (7.3 ± 0.4, *P* < 0.001), filgotinib 100 mg plus MTX (5.8 ± 0.5, *P* < 0.001), and filgotinib 200 mg monotherapy (6.4 ± 0.5, *P* = 0.001) had greater improvements from baseline in SF-36 PCS relative to MTX alone (4.1 ± 0.4) (Table 1). Patients treated with filgotinib 200 mg plus MTX maintained significant improvements relative to MTX alone through week 52, while such improvements remained significant through week 24 for filgotinib 100 mg plus MTX and through week 12 for filgotinib 200 mg monotherapy. Improvements in SF-36 MCS from baseline were greater among the filgotinib 200 mg plus MTX (4.6 ± 0.5, *P* < 0.001), filgotinib 100 mg plus MTX (3.4 ± 0.6, *P* = 0.032), and filgotinib 200 mg monotherapy (4.3 ± 0.6, *P* < 0.001) groups relative to MTX alone (1.9 ± 0.5) at week 4 (Table 1). Thereafter, changes from baseline were similar between the filgotinib groups and the MTX alone group.

At week 4 in MTX-IR patients, improvements from baseline in SF-36 PCS and MCS for patients in the filgotinib 200 mg (6.0 ± 0.5 and 3.3 ± 0.6, respectively; both *P* < 0.001) and 100 mg (5.1 ± 0.5, *P* < 0.001 and 3.0 ± 0.6, *P* = 0.002, respectively) groups were greater compared with placebo (3.4 ± 0.6 and 1.5 ± 0.6); increases were maintained throughout the study (Table 2). MTX-IR patients receiving filgotinib 200 mg reported greater improvements relative to adalimumab at weeks 4 and 12 but not at weeks 24 and 52 (Table 2). Among patients receiving filgotinib 100 mg, improvements in SF-36 PCS and MCS were similar to those in patients receiving adalimumab throughout the study.

Among bDMARD-IR patients, filgotinib 200 mg provided improvements from baseline compared to placebo for SF-36 PCS at weeks 4 (5.9 ± 0.7 vs 3.4 ± 0.7, *P* < 0.001), 12 (8.4 ± 0.8 vs 4.2 ± 0.8, *P* < 0.001), and 24 (9.6 ± 0.8 vs 5.8 ± 0.9, *P* < 0.001) and for SF-36 MCS at weeks 4 (2.5 ± 0.9 vs 0.3 ± 0.9, *P* = 0.019) and 12 (4.5 ± 0.9 vs 2.5 ± 0.9, *P* = 0.045) but not at week 24. Filgotinib 100 mg improved SF-36 PCS relative to placebo at weeks 4 (5.4 ± 0.7 vs 3.4 ± 0.7, *P* = 0.005), 12 (7.6 ± 0.8 vs 4.2 ± 0.8, *P* < 0.001), and 24 (8.9 ± 0.8 vs 5.8 ± 0.9, *P* = 0.002) (Table 3). Improvements in SF-36 MCS were similar for patients receiving filgotinib 100 mg and placebo throughout the study.

### Differences between treatment groups in proportions of patients achieving SF-36 MCID

**Fig. 2:**
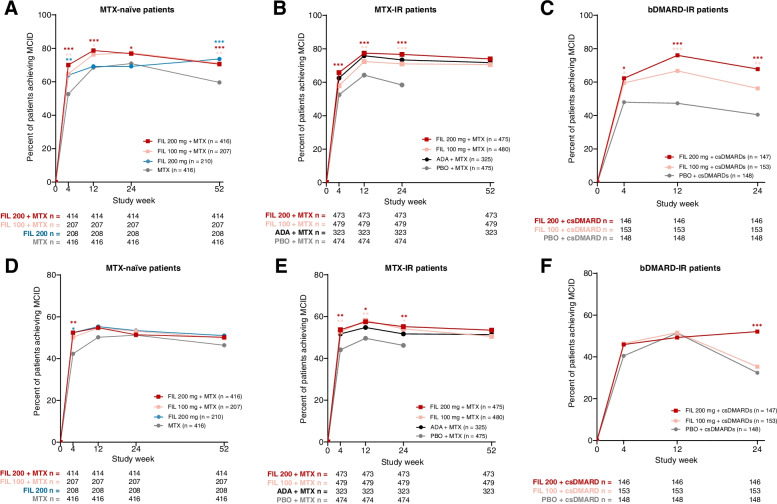
Proportion of patients achieving MCID for SF-36 PCS in **A** MTX-naïve patients, **B** MTX-IR patients, and **C** bDMARD-IR patients; proportion of patients achieving MCID for SF-36 MCS in **D** MTX-naïve, **E** MTX-IR, and **F** bDMARD-IR. Comparison with PBO or MTX: ^***^P < 0.001, ^**^P < 0.01, ^*^P < 0.05. All *P* values are exploratory (not adjusted for multiplicity) and were from logistic regression with treatment groups and stratification factors in the model. A nonresponder imputation was used for patients with missing data. MCID was defined as a ≥ 2.5-point increase from baseline. In the MTX-IR trial, patients on PBO were rerandomized to FIL 200 or 100 mg at week 24. The study in bDMARD-IR patients ended at week 24. ADA, adalimumab; bDMARD, biologic DMARD; csDMARD, conventional synthetic DMARD; DMARD, disease-modifying antirheumatic drug; FIL, filgotinib; MCID, minimal clinically important difference; MCS, mental component score; MTX, methotrexate; PBO, placebo; PCS, physical component score; SF-36, Medical Outcomes Study 36-Item Short Form

At week 4, greater proportions of MTX-naïve patients who received filgotinib 200 mg plus MTX (70.0%, *P* < 0.001), filgotinib 100 mg plus MTX (64.7%, *P* = 0.004), and filgotinib 200 mg monotherapy (63.9%, *P* = 0.006) achieved the MCID for SF-36 PCS compared to MTX alone (52.6%). For filgotinib 200 mg plus MTX compared to MTX alone, significantly greater proportions of patients achieved MCID at weeks 12, 24, and 52; for filgotinib 100 mg plus MTX, the differences were significant at weeks 12 and 52; and for filgotinib 200 mg as monotherapy, the difference from MTX alone was significant at week 52. Greater proportions of patients who received filgotinib 200 mg plus MTX (52.4%, *P* = 0.004), filgotinib 100 mg plus MTX (50.2%, *P* = 0.060), and filgotinib 200 mg monotherapy (52.4%, *P* = 0.016) achieved MCID for SF-36 MCS compared to MTX alone (42.3%) at week 4; at all other timepoints, proportions were similar among filgotinib groups and MTX alone (Fig. 2A, D).

At week 4, 65.8% (*P* < 0.001) of MTX-IR patients who received filgotinib 200 mg and 58.0% (*P* = 0.10) who received filgotinib 100 mg achieved MCID for SF-36 PCS compared with 52.5% of the placebo group; differences between the filgotinib groups and placebo were also significant at weeks 12 and 24. At week 52, 74.0% of patients who received filgotinib 200 mg and 70.6% who received filgotinib 100 mg reached MCID for SF-36 PCS (Fig. 2B). In MTX-IR patients, 53.7% (*P* = 0.002) of the filgotinib 200 and 52.6% (*P* = 0.007) of the filgotinib 100 mg groups achieved MCID for SF-36 MCS compared to 44.1% of the placebo group at week 4 (Fig. 2E); differences remained significant between filgotinib groups and placebo at weeks 12 and 24. The proportions of MTX-IR patients in the filgotinib groups who achieved MCID for SF-36 PCS and MCS were similar to those of the adalimumab group throughout the study.

Among bDMARD-IR patients, the proportion of patients achieving MCID was higher for patients in the filgotinib 200 mg group relative to placebo at weeks 4 (62.3% vs 48.0%, *P* = 0.010), 12 (76.0% vs 47.3%, *P* < 0.001), and 24 (67.8% vs 40.5%, *P* < 0.001) for SF-36 PCS and was similar to placebo until week 24 (52.1% vs 32.4%, *P* < 0.001) for SF-36 MCS (Fig. 2C, F). Among patients who received filgotinib 100 mg compared with placebo, the proportion who achieved MCID for SF-36 PCS was higher at weeks 4 (59.5% vs 48.0%, *P* = 0.049), 12 (66.7% vs 47.3%, *P* < 0.001), and 24 (56.2% vs 40.5%, *P* = 0.007) and was similar to placebo throughout the study for SF-36 MCS.

**Fig. 3:**
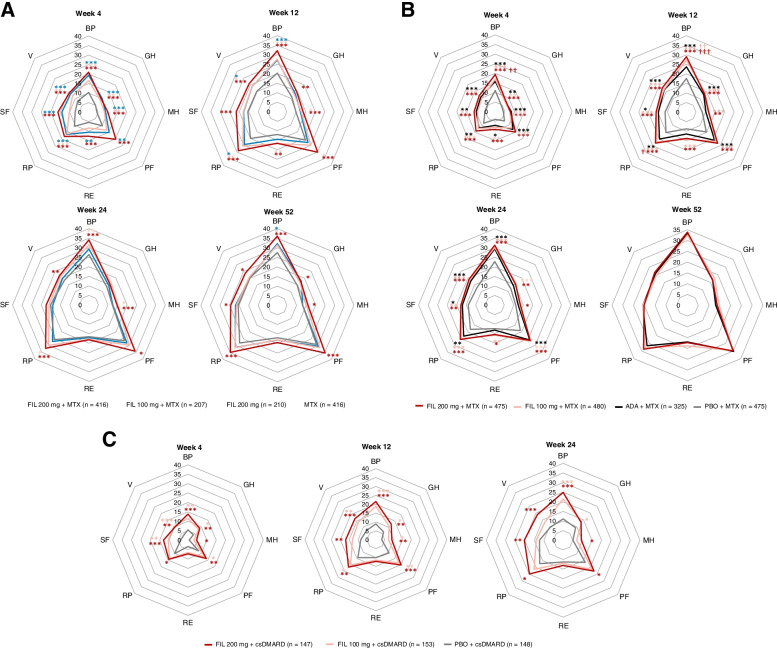
LS mean change from baseline in SF-36 individual domains by visit in **A** MTX-naïve patients, filgotinib dosing regimens compared with MTX: **B** MTX-IR patients, and filgotinib dosing regimens compared with placebo and adalimumab **C** bDMARD-IR patients, filgotinib dosing regimens compared with placebo. Concentric octagons represent the LS mean change from baseline in the SF-36 domain score. Comparison with placebo or MTX: ^***^P < 0.001, ^**^P < 0.01, ^*^P < 0.05. Comparison with ADA: ^†††^*P* < 0.001, ^††^*P* < 0.01. All *P* values are exploratory (not adjusted for multiplicity) and were from the MMRM including treatment, visit (as categorical), treatment by visit, stratification factors, baseline value as fixed effects, and patients being the random effect. In the MTX-IR study, patients receiving PBO were rerandomized to FIL 200 or 100 mg at week 24. The bDMARD-IR study ended at week 24. ADA, adalimumab; BP, bodily pain; bDMARD, biologic DMARD; csDMARD, conventional synthetic DMARD; DMARD, disease-modifying antirheumatic drug; FIL, filgotinib; GH, general health; LS, least-squares; MH, mental health; MMRM, mixed-effects model for repeated measures; MTX, methotrexate; PBO, placebo; PF, physical functioning; R-E, role-emotional; R-P, role-physical; SF, social functioning; SF-36, Medical Outcomes Study 36-Item Short Form; V, vitality

In all 3 studies, improvements from baseline in individual SF-36 domain scores were evident as early as week 4 for MTX-naïve, MTX-IR, and bDMARD-IR patients treated with either dose of filgotinib and persisted throughout treatment until week 52 (Fig. 3).

### Differences between treatment groups in FACIT-Fatigue changes from baseline

At week 4, MTX-naïve patients who received filgotinib 200 or 100 mg plus MTX and filgotinib 200 mg monotherapy reported significantly greater improvements in FACIT-Fatigue compared to MTX alone. Improvements were maintained through week 52 (Table 1). By week 4, MTX-IR patients who received filgotinib 200 or 100 mg demonstrated significant improvements from baseline in FACIT-Fatigue scores relative to placebo as did bDMARD-IR patients who received filgotinib 200 or 100 mg. Improvements were maintained through week 52 and week 24 for MTX-IR and bDMARD-IR patients, respectively (Tables 2 and 3). FACIT-Fatigue scores were numerically greater in MTX-IR patients receiving any dose of filgotinib compared with adalimumab.

### Differences between treatment groups in proportions of patients achieving FACIT-Fatigue MCID

**Fig. 4:**
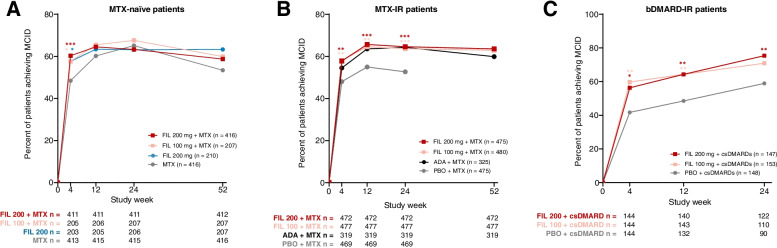
Proportion of patients achieving MCID for FACIT-Fatigue in **A** MTX-naïve patients, **B** MTX-IR patients, and **C** bDMARD-IR patients. Comparison with placebo or methotrexate: ^***^P < 0.001, ^**^P < 0.01, ^*^P < 0.05. Comparison with adalimumab: ^†^*P* < 0.05. All *P* values are exploratory (not adjusted for multiplicity) and were from logistic regression with treatment groups and stratification factors in the model. A nonresponder imputation was used for patients with missing data. MCID was defined as a ≥ 4-point increase from baseline. In the MTX-IR study, patients on PBO were rerandomized to FIL 200 or 100 mg at week 24. The bDMARD-IR study ended at week 24. ADA, adalimumab; bDMARD, biologic DMARD; csDMARD, conventional synthetic DMARD; DMARD, disease-modifying antirheumatic drug; FACIT, Functional Assessment of Chronic Illness Therapy; FIL, filgotinib; MCID, minimal clinically important difference; MTX, methotrexate; PBO, placebo

Among MTX-naïve patients, the MCID for FACIT-Fatigue was achieved by 60.3% of the filgotinib 200 mg plus MTX (*P* < 0.001), 57.6% of the filgotinib 100 mg plus MTX (*P* = 0.025), and 57.6% of the filgotinib 200 mg monotherapy (*P* = 0.033) groups relative to 48.4% of the MTX group (Fig. 4A) at week 4; proportions achieving MCID were similar across groups at weeks 12, 24, and 52. Among MTX-IR patients, at weeks 4 and 24, 57.8% (*P* = 0.002) and 64.6% (*P* < 0.001) of patients who received filgotinib 200 mg and 58.1% (*P* = 0.002) and 63.7% (*P* < 0.001) of patients who received filgotinib 100 mg achieved MCID relative to 48% and 52.7% of the placebo group. The percentage of patients treated with filgotinib who achieved MCID remained consistent through week 52 (Fig. 4B). Results were similar between patients who received either dose of filgotinib and adalimumab throughout the study (Fig. 4B). At week 4, a greater proportion of bDMARD-IR patients who received filgotinib 200 mg (56.3%, *P* = 0.013) and filgotinib 100 mg (59.7%, *P* = 0.003) achieved MCID relative to placebo (41.7%), and at week 24, a greater proportion of patients receiving filgotinib 200 mg (75.4%, *P* = 0.008), but not filgotinib 100 mg, achieved MCID vs placebo (58.9%) (Fig. 4C).

### Differences between treatment groups in WPAI-RA changes from baseline

At baseline, the percentage of employed MTX-naïve patients in the filgotinib 200 mg plus MTX, filgotinib 100 mg plus MTX, filgotinib 200 mg monotherapy, and MTX groups were 42.3%, 39.6%, 44.7%, and 41.2%, respectively. At week 4, least-squares mean change from baseline was significantly greater for filgotinib 200 and 100 mg plus MTX and for filgotinib monotherapy relative to MTX monotherapy for presenteeism, work productivity loss, and activity impairment. Changes in absenteeism were similar between patients receiving any dose of filgotinib and MTX alone. Improvements were maintained through week 52 (Table 1).

At baseline, the percentage of employed MTX-IR patients in the filgotinib 200 mg, filgotinib 100 mg, adalimumab, and placebo groups were 42.8%, 41.5%, 41.1%, and 35.6%, respectively. Patients in the filgotinib 200 mg group reported significantly greater improvements relative to placebo at weeks 4, 12, and 24 in presenteeism, work productivity loss, and activity impairment. Improvements in absenteeism were similar between patients receiving filgotinib 200 mg and placebo at weeks 4 and 12 but greater at week 24 for patients receiving filgotinib 200 mg (Table 2).

Among MTX-IR patients receiving filgotinib 100 mg, improvements relative to placebo were similar at week 4 but significantly greater at weeks 12 and 24 for presenteeism and work productivity loss. Improvements were significantly greater for patients receiving filgotinib 100 mg relative to placebo at weeks 4, 12, and 24 for activity impairment. For absenteeism, improvements were similar between patients receiving filgotinib 100 mg and placebo at weeks 4 and 12 but greater at week 24 for patients receiving filgotinib 100 mg (Table 2). Improvements in presenteeism, work productivity loss, and activity impairment were maintained through 52 weeks (Table 2). Results were similar compared with adalimumab.

At baseline, the percentages of employed bDMARD-IR patients receiving filgotinib 200 mg (24.0%), filgotinib 100 mg (35.5%), and placebo (36.1%) were lower compared to the MTX-IR and MTX-naïve study populations. Among bDMARD-IR patients who received filgotinib 200 mg, absenteeism was similar to patients receiving placebo at weeks 4 and 24 but significantly improved relative to placebo at week 12. Improvements for patients receiving filgotinib 200 mg relative to placebo were significantly greater at weeks 4 and 12 for presenteeism and work productivity but not at week 24. Activity impairment was significantly improved relative to placebo at weeks 4, 12, and 24 (Table 3). For bDMARD-IR patients who received filgotinib 100 mg, presenteeism and work productivity loss were significantly improved relative to placebo at week 12, but improvements were similar at weeks 4 and 24. Activity impairment was significantly improved at weeks 4, 12, and 24 (Table 3).

### Differences between treatment groups in PtGA changes from baseline

In all 3 studies, MTX-naïve, MTX-IR, and bDMARD-IR patients experienced rapid and sustained improvements in patient-assessed disease activity (Tables 1, 2, and 3). MTX-naïve patients who received filgotinib reported significantly greater improvements in PtGA at week 4 relative to MTX monotherapy, and improvements continued and were maintained through week 52 (Table 1). Similarly, MTX-IR patients who received filgotinib reported significantly greater improvements relative to placebo at weeks 4, 12, and 24 (Table 2). With the exception of a significantly greater reduction from baseline among patients receiving filgotinib 200 mg relative to adalimumab at week 12, improvements in PtGA were similar for either dose of filgotinib and adalimumab throughout the study. bDMARD-IR patients who received filgotinib 200 mg and filgotinib 100 mg reported significant improvements relative to placebo at all timepoints (Table 3).

## Discussion

Once-daily filgotinib 200 or 100 mg in combination with MTX or with csDMARDs provided rapid and sustained improvements in functional status, HRQL, fatigue, presenteeism, work productivity, and disease activity as reported by patients with RA who were MTX-naïve or who had inadequate response to MTX or bDMARDs. Filgotinib in combination with MTX provided improvements at levels similar to or better than adalimumab plus MTX for patients with an inadequate response to MTX for up to 52 weeks. Improvements in functional status, fatigue, and physical HRQL were beyond the minimally important differences for many patients, and a higher proportion experienced minimally important differences with either dosage of filgotinib compared with placebo. In general, in these exploratory analyses, therapy with filgotinib 200 mg plus MTX or csDMARDs appeared to provide a greater benefit in each PRO measured for MTX-naïve, MTX-IR, and bDMARD-IR patients. The results of this analysis were similar to those observed in other Phase 3 trials of the JAK inhibitors tofacitinib, baricitinib, and upadacitinib [16–18, 21–26].

A key strength of this analysis is the inclusion of 3 separate clinical trials that evaluated filgotinib in different patient populations. This allowed us to better evaluate the effect of filgotinib in an MTX-naïve patient population relative to patients with inadequate response to csDMARDs (MTX) or bDMARDs. Limitations of the study include that all analyses and *P* values, with the exception of HAQ-DI mean change from baseline at the primary timepoints, were exploratory. Accordingly, although improvements relative to the placebo or active controls were reported, the statistical significance of the changes should be interpreted with caution. Second, although 3 patient populations were included, statistical comparisons of filgotinib treatment were not performed between studies, and any conclusions regarding the relative effectiveness of filgotinib in one patient population vs another are conjectural. Third, the bDMARD-IR trial was limited in duration to 24 weeks and enrolled a smaller number of patients, which precludes conclusions regarding duration of benefit among bDMARD-IR patients.

## Conclusions

Filgotinib improved patient functional status, HRQL, fatigue, work impairment, and assessments of disease activity in multiple patient populations. These PRO results suggest filgotinib used as monotherapy or in combination can be an effective treatment option for patients with insufficient response to MTX or bDMARDs, as well as for patients who are MTX-naïve. Additional analyses of the different RA patient populations with long-term treatment will better define the role of filgotinib in improving HRQL in MTX-naïve and in treatment-refractory populations. An extension trial (FINCH 4, NCT03025308) to evaluate the long-term outcomes of patients who completed these 3 studies is ongoing.

## Supplementary Information

**Additional file 1: Supplementary Table 1.** Patient demographics and baseline characteristics, MTX-naïve trial.

**Additional file 2: Supplementary Table 2.** Patient demographics and baseline characteristics, MTX-IR trial.

**Additional file 3: Supplementary Table 3.** Patient demographics and baseline characteristics, bDMARD-IR trial.

## Data Availability

Data are available upon reasonable request. Anonymized individual patient data will be shared upon request for research purposes dependent upon the nature of the request, the merit of the proposed research, the availability of the data, and the intended use. The full data sharing policy for Gilead Sciences can be found at https://www.gilead.com/about/ethics-and-code-of-conduct/policies.

## Abbreviations

ACR: American College of Rheumatology
bDMARD-IR: Biologic disease-modifying antirheumatic drug
csDMARD: Conventional synthetic disease-modifying antirheumatic drug
FACIT-Fatigue: Functional Assessment of Chronic Illness Therapy-Fatigue
HAQ-DI: Health Assessment Questionnaire-Disability Index
HRQL: Health-related quality of life
IR: Inadequate response
JAK1: Janus kinase 1
MCID: Minimal clinically important difference
MCS: Mental Component Summary (SF-36)
MMRM: Mixed-effects model for repeated measures
MTX-IR: Methotrexate
PCS: Physical Component Summary (SF-36)
PRO: Patient-reported outcome
PtGA: Patient Global Assessment of Disease Activity
RA: Rheumatoid arthritis
SF-36: Medical Outcomes Study 36-Item Short Form
SE: Standard error
WPAI-RA: Work Productivity and Activity Impairment Questionnaire-Rheumatoid Arthritis
