# Supplementary material for: The impact of filgotinib on patient-reported outcomes and health-related quality of life for patients with active rheumatoid arthritis: a post hoc analysis of Phase 3 studies
Source: Arthritis Res Ther. 2022 Jan 3;24:11. doi: 10.1186/s13075-021-02677-7 (PMC8722138; doi:10.1186/s13075-021-02677-7)
Supplement: Supplementary file 1 — Additional file 1: Supplementary Table 1. Patient demographics and baseline characteristics, MTX-naïve trial. [file 13075_2021_2677_MOESM1_ESM.docx]

**Supplementary Table 1.** Patient demographics and baseline characteristics, MTX-naïve trial

| **Characteristic** | **FIL 200 mg**  **+ MTX**  **n = 416** | **FIL 100 mg**  **+ MTX**  **n = 207** | **FIL 200 mg**  **n = 210** | **MTX**  **n = 416** |
| --- | --- | --- | --- | --- |
| **Age** (years), median (range) | 53 (18, 85) | 54 (19, 79) | 52 (18, 81) | 53 (18, 87) |
| **Duration of RA from diagnosis** (years), median (range) | 0.4 (0, 26.8) | 0.4 (0, 31.7) | 0.4 (0,47.6) | 0.3 (0, 52.3) |
| **Presence of RF and anti-CCP**, n (%) | 252 (60.6) | 122 (58.9) | 112 (53.3) | 258 (62.0) |
| **SJC66**, mean (SD) | 16 (9.8) | 16 (9.3) | 16 (9.7) | 16 (9.4) |
| **TJC68**, mean (SD) | 26 (14.5) | 25 (13.9) | 26 (13.7) | 26 (13.8) |
| **DAS28(CRP),** mean (SD) | 5.7 (1.0) | 5.7 (1.0) | 5.8 (0.9) | 5.7 (1.0) |
| **HAQ-DI**, mean (SD) | 1.52 (0.6) | 1.56 (0.7) | 1.56 (0.7) | 1.60 (0.6) |
| **SF-36**, mean (SD) |  |  |  |  |
| PCS | 33.9 (7.5) | 33.7 (8.0) | 33.6 (7.7) | 33.3 (7.3) |
| MCS | 44.6 (10.6) | 43.2 (11.5) | 43.1 (11.3) | 43.5 (11.5) |
| Bodily pain | 33.6 (16.8) | 32.6 (18.3) | 32.6 (18.2) | 31.5 (15.8) |
| General health | 43.7 (18.1) | 42.6 (19.6) | 40.6 (16.1) | 42.1 (17.3) |
| Mental health | 58.4 (19.4) | 55.4 (20.8) | 56.7 (20.1) | 56.3 (20.8) |
| Physical functioning | 36.4 (25.4) | 34.6 (26.4) | 35.3 (25.9) | 34.6 (25.6) |
| Role-emotional | 62.0 (27.2) | 57.8 (26.3) | 56.9 (28.5) | 59.5 (28.4) |
| Role-physical | 39.6 (21.5) | 36.8 (22.9) | 37.7 (23.1) | 36.7 (22.8) |
| Social functioning | 56.6 (25.1) | 55.4 (25.6) | 54.4 (25.1) | 53.8 (25.6) |
| Vitality | 39.3 (19.0) | 39.2 (20.6) | 38.4 (19.6) | 38.1 (19.2) |
| **FACIT-F**, mean (SD) | 28.3 (10.9) | 27.3 (11.9) | 27.3 (10.9) | 27.1 (10.7) |
| **PtGA**, mean (SD) | 65 (21.0) | 66 (21.6) | 68 (19.2) | 66 (21.0) |
| **CDAI**, mean (SD) | 39.5 (11.8) | 39.2 (12.7) | 40.0 (12.6) | 40.2 (12.5) |

anti-CCP, anti-cyclic citrullinated peptide; CDAI, Clinical Disease Activity Index; DAS28(CRP), Disease Activity Score 28 with C-reactive protein; FACIT-F, functional assessment of chronic illness therapy-fatigue; FIL, filgotinib; HAQ-DI, Health Assessment Questionnaire-Disability Index; MCS, Mental Component Score; MTX, methotrexate; PCS, Physical Component Score; PtGA, Patient Global Assessment of Disease Activity; RA, rheumatoid arthritis; RF, rheumatoid factor; SD, standard deviation; SF-36, Medical Outcomes Study 36-Item Short Form; SJC66, swollen joint count based on 66 joints; TJC68, tender joint count based on 68 joints.
