# Supplementary material for: The impact of filgotinib on patient-reported outcomes and health-related quality of life for patients with active rheumatoid arthritis: a post hoc analysis of Phase 3 studies
Source: Arthritis Res Ther. 2022 Jan 3;24:11. doi: 10.1186/s13075-021-02677-7 (PMC8722138; doi:10.1186/s13075-021-02677-7)
Supplement: Supplementary file 2 — Additional file 2: Supplementary Table 2. Patient demographics and baseline characteristics, MTX-IR trial. [file 13075_2021_2677_MOESM2_ESM.docx]

**Supplementary Table 2.** Patient demographics and baseline characteristics, MTX-IR trial

| **Characteristic** | **FIL 200 mg + MTX**  **n = 475** | **FIL 100 mg +**  **MTX**  **n = 480** | **ADA + MTX**  **n = 325** | **PBO + MTX**  **n = 475** |
| --- | --- | --- | --- | --- |
| **Age** (years), median (range) | 53 (18, 81) | 54 (19, 86) | 55 (18, 81) | 54 (19, 84) |
| **Duration of RA from diagnosis** (years), median (range) | 4.8 (0.3, 45.4) | 5.8 (0.1, 41.5) | 5.5 (0.3, 37.9) | 5.1 (0.1, 46.6) |
| **Presence of RF or anti-CCP**, n (%) | 401 (84.4) | 411 (85.6) | 275 (84.6) | 410 (86.3) |
| **SJC66**, mean (SD) | 15 (8.5) | 15 (8.5) | 16 (8.4) | 16 (8.5) |
| **TJC68**, mean (SD) | 25 (13.5) | 25 (13.4) | 24 (13.2) | 24 (13.5) |
| **DAS28(CRP),** mean (SD) | 5.8 (0.9) | 5.7 (1.0) | 5.7 (0.9) | 5.7 (0.9) |
| **HAQ-DI**, mean (SD) | 1.59 (0.61) | 1.55 (0.63) | 1.59 (0.60) | 1.63 (0.61) |
| **SF-36**, mean (SD) |  |  |  |  |
| PCS | 33.4 (7.2) | 33.6 (7.8) | 32.8 (7.7) | 32.9 (7.1) |
| MCS | 43.9 (10.4) | 44.6 (10.4) | 44.1 (10.4) | 43.4 (11.0) |
| Bodily pain | 33.1 (16.5) | 33.4 (16.4) | 31.4 (16.3) | 31.1 (15.5) |
| General health | 39.2 (16.9) | 39.9 (17.5) | 38.0 (16.9) | 38.7 (16.7) |
| Mental health | 56.3 (18.7) | 57.6 (18.9) | 56.4 (18.4) | 55.9 (19.5) |
| Physical functioning | 34.4 (22.8) | 35.9 (24.7) | 34.2 (23.8) | 33.8 (23.5) |
| Role-emotional | 59.2 (26.5) | 60.8 (26.8) | 60.9 (25.5) | 58.7 (27.7) |
| Role-physical | 39.0 (21.2) | 39.6 (22.7) | 38.5 (21.8) | 37.6 (20.9) |
| Social functioning | 56.8 (24.5) | 57.9 (23.7) | 55.7 (24.0) | 54.2 (23.7) |
| Vitality | 40.4 (18.5) | 41.5 (19.0) | 38.6 (17.6) | 38.4 (17.5) |
| **FACIT-F**, mean (SD) | 27.6 (10.7) | 27.8 (10.6) | 27.2 (10.2) | 26.9 (10.3) |
| **PtGA**, mean (SD) | 67 (19.2) | 65 (19.7) | 67 (19.1) | 68 (18.7) |
| **CDAI**, mean (SD) | 39.5 (11.9) | 38.6 (12.2) | 39.2 (11.5) | 39.6 (11.7) |

ADA, adalimumab; anti-CCP, anti-cyclic citrullinated peptide; CDAI, Clinical Disease Activity Index; DAS28(CRP), Disease Activity Score with C-reactive protein; FACIT-F, functional assessment of chronic illness therapy-fatigue; FIL, filgotinib; HAQ-DI, Health Assessment Questionnaire-Disability Index; MCS, Mental Component Score; MTX, methotrexate; PBO, placebo; PCS, Physical Component Score; PtGA, Patient Global Assessment of Disease Activity; RA, rheumatoid arthritis; RF, rheumatoid factor; SD, standard deviation; SF-36, Medical Outcomes Study 36-Item Short Form; SJC66, swollen joint count based on 66 joints; TJC68, tender joint count based on 68 joints.
