# Supplementary material for: The impact of filgotinib on patient-reported outcomes and health-related quality of life for patients with active rheumatoid arthritis: a post hoc analysis of Phase 3 studies
Source: Arthritis Res Ther. 2022 Jan 3;24:11. doi: 10.1186/s13075-021-02677-7 (PMC8722138; doi:10.1186/s13075-021-02677-7)
Supplement: Supplementary file 3 — Additional file 3: Supplementary Table 3. Patient demographics and baseline characteristics, bDMARD-IR trial. [file 13075_2021_2677_MOESM3_ESM.docx]

**Supplementary Table 3.** Patient demographics and baseline characteristics, bDMARD-IR trial

|  | **FIL 200 mg**  **+ csDMARD**  **n = 147** | **FIL 100 mg**  **+ csDMARD**  **n = 153** | **PBO  + csDMARD**  **n = 148** |
| --- | --- | --- | --- |
| **Age, years,** median (range) | 56 (20, 80) | 56 (25, 80) | 57 (25, 83) |
| **Duration of RA from diagnosis, years,** mean (SD) | 12.6 (9.5) | 12.0 (7.7) | 12.6 (10.3) |
| **Presence of RF or anti-CCP** | 112 (76.2) | 118 (77.1) | 113 (76.4) |
| **Number of prior bDMARDs** |  |  |  |
| <3 | 109 (74.1) | 119 (77.8) | 114 (77.0) |
| **Number of concurrent csDMARDs** |  |  |  |
| 0 | 0 | 0 | 1 (0.7) |
| 1 | 133 (90.5) | 135 (88.2) | 135 (91.2) |
| 2 | 14 (9.5) | 18 (11.8) | 12 (8.1) |
| **SJC66**, mean (SD) | 18 (12.5) | 17 (12.4) | 17 (9.7) |
| **TJC68**, mean (SD) | 28 (16.1) | 26 (15.4) | 27 (15.5) |
| **DAS28(CRP),** mean (SD) | 5.9 (1.0) | 5.9 (1.0) | 5.9 (0.9) |
| **HAQ-DI,** mean (SD) | 1.70 (0.7) | 1.64 (0.7) | 1.65 (0.6) |
| **SF-36** mean (SD) |  |  |  |
| PCS | 30.4 (7.8) | 31.7 (7.8) | 31.1 (8.2) |
| MCS | 44.5 (12.0) | 44.2 (11.6) | 44.3 (11.3) |
| Bodily pain | 29.7 (17.9) | 30.3 (17.2) | 28.9 (17.1) |
| General health | 38.2 (16.7) | 37.9 (19.0) | 38.6 (16.2) |
| Mental health | 58.5 (21.9) | 59.1 (20.3) | 59.5 (21.0) |
| Physical functioning | 27.6 (22.3) | 31.3 (25.5) | 29.7 (25.1) |
| Role-emotional | 61.2 (29.7) | 60.5 (29.4) | 57.6 (28.5) |
| Role-physical | 32.4 (23.8) | 37.5 (24.8) | 33.8 (23.8) |
| Social functioning | 51.9 (27.4) | 53.0 (27.6) | 51.9 (24.8) |
| Vitality | 32.5 (18.3) | 34.3 (19.9) | 36.3 (19.5) |
| **FACIT-F,** mean (SD) | 24.2 (11.5) | 23.7 (12.3) | 25.4 (10.9) |
| **PtGA,** mm, mean (SD) | 68 (20.6) | 69 (20.2) | 70 (18.0) |
| **CDAI,** mean (SD) | 41.7 (14.2) | 40.4 (13.2) | 41.4 (12.0) |

Data presented as n (%) unless otherwise specified.

bDMARD, biologic DMARD; anti-CCP, anti-cyclic citrullinated peptide; CDAI, Clinical Disease Activity Index; csDMARD, conventional synthetic DMARD; DAS28(CRP), Disease Activity Score 28 with C-reactive protein; DMARD, disease-modifying antirheumatic drug; FACIT-F, Functional Assessment of Chronic Illness Therapy-Fatigue; FIL, filgotinib; HAQ-DI, Health Assessment Questionnaire-Disability Index; MCS, Mental Component Score; PBO, placebo; PCS, Physical Component Score; PtGA, Patient Global Assessment of Disease Activity; RA, rheumatoid arthritis; RF, rheumatoid factor, SD, standard deviation; SF-36, Medical Outcomes Study 36-Item Short Form; SJC66, swollen joint count based on 66 joints; TJC68, tender joint count based on 68 joints.
